# Application of CRISPR/Cas9 Nuclease in Amphioxus Genome Editing

**DOI:** 10.3390/genes11111311

**Published:** 2020-11-05

**Authors:** Liuru Su, Chenggang Shi, Xin Huang, Yiquan Wang, Guang Li

**Affiliations:** State Key Laboratory of Cellular Stress Biology, School of Life Sciences, Xiamen University, Xiangan District, Xiamen 361102, Fujian, China; 18059054571@163.com (L.S.); chenggangstone@163.com (C.S.); hx18359276362@163.com (X.H.); wangyq@xmu.edu.cn (Y.W.)

**Keywords:** amphioxus, CRISPR/Cas9, genome editing, knockdown

## Abstract

The cephalochordate amphioxus is a promising animal model for studying the origin of vertebrates due to its key phylogenetic position among chordates. Although transcription activator-like effector nucleases (TALENs) have been adopted in amphioxus genome editing, its labor-intensive construction of TALEN proteins limits its usage in many laboratories. Here we reported an application of the CRISPR/Cas9 system, a more amenable genome editing method, in this group of animals. Our data showed that while co-injection of *Cas9* mRNAs and sgRNAs into amphioxus unfertilized eggs caused no detectable mutations at targeted loci, injections of *Cas9* mRNAs and sgRNAs at the two-cell stage, or of Cas9 protein and sgRNAs before fertilization, can execute efficient disruptions of targeted genes. Among the nine tested sgRNAs (targeting five genes) co-injected with Cas9 protein, seven introduced mutations with efficiency ranging from 18.4% to 90% and four caused specific phenotypes in the injected embryos. We also demonstrated that monomerization of sgRNAs via thermal treatment or modifying the sgRNA structure could increase mutation efficacies. Our study will not only promote application of genome editing method in amphioxus research, but also provide valuable experiences for other organisms in which the CRISPR/Cas9 system has not been successfully applied.

## 1. Introduction

The cephalochordate amphioxus represents a transitional group between invertebrates and vertebrates, showing most similarities with vertebrates in terms of either body plan, genome structure or embryogenesis among living animals. Compared to vertebrates, amphioxus has not undergone extensive genome duplications [1] and lacks paired eyes, bones, appendages, a sophisticated brain and most visceral organs. Because of these characteristics, amphioxus has been thought to be a promising model for studying the origin and evolution of vertebrate complexity [2]. To develop amphioxus as a model organism, several breakthroughs have recently been made, which include development of methods for manipulating amphioxus into spawning on demand throughout the year [3,4] and generating amphioxus mutants with the TALEN system [5] and transgenes via the *Tol2* transposon system [6]. In the present study, we reported a highly efficient, more amenable genome editing method in this organism, using the clustered regularly interspaced short palindromic repeat/Cas9 (CRISPR/Cas9) system [7].

## 2. Materials and Methods

### 2.1. Amphioxus Maintenance and Image Acquisition

Amphioxus *Branchiostoma floridae* were obtained from Dr. Jr-Kai Yu’s laboratory at Institute of Cellular and Organismic Biology, Academia Sinica, Taiwan. They were maintained and induced to spawn by essentially following the protocols as we described and used in *Branchiostoma. belcheri* animals [3,4], but slightly differently from the one developed recently for *B. floridae* [8]. The amphioxus transgenic line Tg (*mylz2:mCherry*) (hemizygous) has been reported previously [6]. Larvae were photographed under an IX71 microscope (Olympus, Tokyo, Japan) or a M165FC stereoscope (Leica, Wetzlar, Germany).

### 2.2. In Vitro Cas9 mRNA Synthesis

pXT7-Cas9 [9] was a gift from China Zebrafish Resource Center, CZRC, Wuhan, China, and pCS2-nls-zCas9-nls [10] was obtained from Dr. Wenbiao Chen. They were linearized using *Xba*I and *Not*I, respectively, purified with the phenol-chloroform method, and used as templates to synthesize *Cas9* mRNA with the mMESSAGE mMACHINE T7 or SP6 Transcription Kits (Thermo Fisher Scientific, Waltham, MA, USA). Synthesized mRNAs were treated with Tango DNase supplemented by the kit, purified using the phenol-chloroform method, dissolved in RNase-free water and stored at −80 °C for use.

### 2.3. sgRNA Design and Synthesis

All sgRNAs except *mCherry*-sgRNA were designed at the website (https://www.crisprscan.org/?page=sequence) according to roles used for zebrafish. Two different versions of gDNA backbones were tested in the study. Version 1 is a regularly used one [11], while version 2 was modified from version 1 by including a 5 bp duplex extension and a T-to-C mutation at position 4 of the continuous sequence of thymines [12]. Version 1 gDNAs (DNA template used to synthesize sgRNA) were generated as follows: for each target site, a pair of primers containing the target sequence and overhang were synthesized, annealed and ligated between two *BsmB*I sites of the pT7-gRNA vector obtained from Dr. Wenbiao Chen [10]. Resultant plasmids were used to amplify version 1 gDNAs with primers M13R, 5′-agcggataacaatttcacacagg-3′; pT7-sgRNA-R1, 5′-gatccgcaccgactcggtgccact-3′. Version 2 gDNAs were obtained with a more straightforward way by annealing a specific oligonucleotide containing the T7 promoter, spacer (target sequence) and partial repeat sequence with a universal oligonucleotide containing a whole repeat sequence. Both version 1 and 2 gDNAs were purified using the phenol-chloroform method before being used as templates to synthesize sgRNAs with the MEGAshortscript™ T7 Transcription Kit (Thermo Fisher Scientific). The synthesized sgRNAs were treated further with Tango DNase, purified using the phenol-chloroform method, resolved in RNase-free water and stored at −80 °C for use. Primers and oligonucleotides used are listed in Supplementary Table S1, and sequence information of sgRNA backbones is provided in Supplementary Table S2.

### 2.4. Injection Solution Preparation and Microinjection

*Cas9* mRNA/sgRNA injection solution was prepared with 5 mg/mL fluorescein-labeled dextrans (10,000 MW, Thermo Fisher Scientific), 20% glycerol, 376.7 μg/μL *Cas9* mRNA and 178.6 μg/μL sgRNA. Cas9 nuclease/sgRNA injection solution was prepared to contain 3 mg/mL fluorescein-labeled dextrans, 12.5% glycerol (not including the glycerol from Cas9 nuclease) and the desired concentration of Cas9 protein and sgRNA. Three Cas9 nucleases which were purchased from New England BioLabs (20 μM, equal to around 3 μg/μL), Thermo Fisher Scientific (1 μg/μL) and TaKaRa (3 μg/μL) were tested in the study. sgRNA was used directly or predenatured as previously described [12]. Before injection, the Cas9 nuclease/sgRNA injection solution was incubated at 37 °C for 5 min in a thermal cycler to facilitate Cas9/sgRNA RNP complex formation. Microinjection of unfertilized eggs was carried out as previously described [13], while single blastomere injection at the two-cell stage was conducted as recently described [14]. Around 100–200 unfertilized eggs or 50 two-cell embryos were injected for each experiment.

### 2.5. Mutant Efficiency Estimation

Genomic DNA of embryos (~20) at and after the 32-cell stage were extracted using an Animal Tissue Direct PCR kit (FOREGENE, Chengdu, China) and those (~200) of unfertilized eggs and two-cell embryos were purified using a DNeasy^®^ Blood & Tissue Kit (Qiagen, Hilden, Germany). DNA fragments flanking target sites were amplified using primers listed in Supplementary Table S3. Mutations were detected using either a T7EI cleavage assay or a restriction enzyme digestion assay. For some experiments, mutations were also confirmed by DNA sequencing. In the T7EI cleavage assay, 20–40 ng amplicons were melted and reannealed in a 10 μL reaction system as follows: 95 °C for 5 min, 95 °C to 20 °C ramping at 0.1 °C/s and holding at 20 °C, then digested by 2U of T7 endonuclease I (T7E1, New England BioLabs, Ipswich, MA, USA) at 37 °C for 15 min; in the restriction enzyme digestion assay, 20–40 ng amplicons were digested in a 10 μL reaction system with the proper enzymes. The digested samples were then subjected to electrophoresis on a 2% agarose gel. Mutation efficiencies were either estimated by comparing band intensity between uncut and cut bands (in the restriction enzyme digestion assay), or calculated using the formula % mutation = 100 × (1-(1-fraction cleaved)^1/2^) [15]. Quantification of band intensity was executed with software implemented in the Tanon Gis system (Tanon, Shanghai, China).

### 2.6. Quantitative RT-PCR

Injection solutions containing 511 ng/μL *mCherry*-sgRNA and 302 ng/μL *Cas9* mRNA, or 511 ng/μL *mCherry*-sgRNA and 906 ng/μL TaKaRa Cas9 protein were used in this experiment. After injection, thirty eggs or embryos were collected at unfertilized egg, two-cell and early gastrula stages. Their total RNAs were then extracted using the TRIzol reagent and reverse-transcribed to cDNA using an Evo M-MLV One Step RT-PCR Kit (Accurate Biotechnology (Hunan) Co., Changsha, China). Real-time quantitative PCR (RT-qPCR) analysis was performed using a TransStart Tip Green qPCR SuperMix kit (TransGen Co., Beijing, China) on a Stratagene Mx3000P real-time PCR system (Stratagene, La Jolla, CA, USA) under the conditions of 94 °C for 30s, 40 cycles at 94 °C for 5 s, 58 °C (for *Cas9* and *Gapdh*) or 61 °C (for *Gapdh* and sgRNA) for 15 s, and 72 °C for 10 s. Expression levels of sgRNA and *Cas9* were normalized to that of *Gapdh* (glyceraldehyde-3-phosphate dehydrogenase), and the 2-^△△^Ct method was used to calculate the relative expression of the two examined genes. Three independent experiments were conducted and their results were used to calculate the averages and standard errors. The primer sequences used are listed in Supplementary Table S4. 

## 3. Results and Discussion

### 3.1. Injection of Cas9 mRNA and sgRNA at 2-Cell Stage, but Not at Unfertilized Egg Stage, Induces Targeted Mutations in Amphioxus

**Figure 1:**
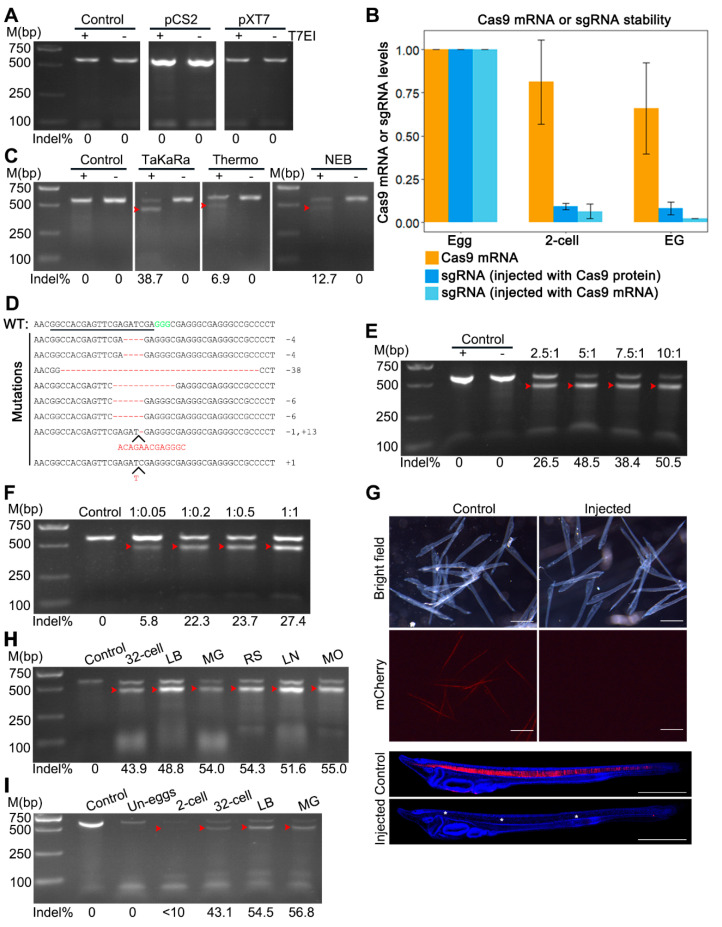
Detection of CRISPR/Cas9-induced mutations at the *mCherry* locus in *mylz2-mCherry* transgenic amphioxus. (**A**) T7EI cleavage assay showing no detectable mutation in uninjected embryos (control), or embryos injected with *mCherry*-sgRNA and *Cas9* mRNA transcribed from pXT7-Cas9 (pXT7) or pCS2-nls-zCas9-nls (pCS2) plasmids. —means no T7 endonuclease I was added and + means T7 endonuclease I was added. (**B**) RT-qPCR analysis of *Cas9* mRNA and *mCherry*-sgRNA expression in embryos injected with Cas9 protein and *mCherry*-sgRNA or *Cas9* mRNA and *mCherry*-sgRNA. Samples were collected and examined at unfertilized egg (egg), two-cell (2-cell) and early gastrula (EG) stages. (**C**) T7EI cleavage assay showing no detectable mutation in uninjected embryos (control) and different levels of mutations in embryos injected, respectively, with *mCherry*-sgRNA and each of the three commercial Cas9 protein (TaKaRa, Thermo and NEB). —means no T7 endonuclease I was added and + means T7 endonuclease I was added. (**D**) Mutations were detected using DNA sequencing in the injected embryos. The wild-type (WT) reference sequence is shown on the top. The underlined sequence is the target site and GGG (green) is the PAM sequence. Deletion is shown by the dashed line and insertion is highlighted by inserted letters. Indels (+, insertion; –, deletion) are listed on the right of each allele. (**E**) Mutation efficiencies in embryos injected with different molar ratios of TaKaRa Cas9 to *mCherry*-sgRNA in which the amount of Cas9 protein was kept constant while that of *mCherry*-sgRNA was different. (**F**) Mutation efficiencies in embryos injected with different molar ratios of TaKaRa Cas9 to *mCherry*-sgRNA in which amount of *mCherry*-sgRNA was kept constant while that of Cas9 protein was different. Red arrowheads in panels C, E, F, H and I mark bands released by T7 endonuclease I digestion. (**G**) Observation of red fluorescent signal in two-day transgenetic larvae. In contrast to uninjected (control) larvae which showed a specific fluorescent signal in the notochord and somites, the injected embryos showed no detectable fluorescent signals. The blue fluorescent signal shows the amphioxus cell nuclei stained with DAPI. Scale bars in all images are 100 μm. (**H**,**I**) Mutation efficiencies in different stages of embryos injected with TaKaRa Cas9 and *mCherry*-sgRNA. Un-eggs, unfertilized eggs; LB, late blastula; MG, mid-gastrula; RS, rotation stage; LM, late neurula; MO, mouth-opening stage.

We first tried injecting amphioxus eggs with *Cas9* mRNA and sgRNA as it was described in other species. Two versions of *Cas9* mRNA from pXT7-hCas9 and pCS2-nls-zCas9-nls vectors which have been shown to be highly efficient in zebrafish [9,10], and a previously reported sgRNA targeting *mCherry* gene (hereafter, *mCherry*-sgRNA) (Figure S1A) [16] were tested. After mixing each of these *Cas9* mRNAs with *mCherry*-sgRNA, we injected them, respectively, into unfertilized eggs of hemizygous *mylz2*:*mCherry* transgenic amphioxus [6]. Unexpectedly, we found no detectable mutations with the T7EI cleavage assay (Figure 1A) and DNA sequencing at the target site for either injection.

We speculated that this negative result might be caused by degradation of sgRNA in amphioxus embryos, since lower efficacy, led by crRNA degradation, has been reported in the application of the Cpf1 system in zebrafish embryos [17]. To test this, we monitored the *mCherry*-sgRNA level at three different stages of embryos using RT-qPCR. Consistent with our speculations, we observed that the *mCherry*-sgRNA level decreased rapidly after injection and only about six percent of embryos were retained at the two-cell stage (about 50 min after injection). The level further reduced to around 2% at the early gastrula stage (Figure 1B). To avoid the major sgRNA decay occurring between the unfertilized egg and two-cell stages, we injected *Cas9* mRNAs and sgRNAs into one blastomere at the two-cell stage. Four sgRNAs were tested and three of them (except *mCherry*-sgRNA) induced mutations at their target sites with efficacies ranging from 21% to 33.3% (Figure S1C). These results demonstrated that sgRNA decay that occurred before the two-cell stage is a major cause for the ineffectiveness of co-injection of *Cas9* mRNAs and sgRNA at the unfertilized egg stage.

### 3.2. Effective Mutations Caused by Injecting Cas9/sgRNA Ribonucleoprotein (RNP) Complexes in Amphioxus Unfertilized Eggs

Since injection of amphioxus embryos at the two-cell stage was much more difficult and often led to more embryo deformation than injection of amphioxus unfertilized eggs, we tried to inject Cas9/sgRNA ribonucleoprotein (RNP) complexes at the unfertilized egg stage. We speculated that the incorporation of sgRNA into Cas9 protein would prevent or at least slow down sgRNA decay observed in the above-mentioned experiments. We tested three kinds of commercial Cas9 nucleases which were purchased, respectively, from TaKaRa, Thermo and NEB companies, at the *mCherry*-sgRNA target site. We detected mutations in embryos injected with each of the three Cas9 nucleases using the T7EI cleavage assay and DNA sequencing (Figure 1C and Figure S1B). Among them, the nuclease from TaKaRa showed the highest mutation efficacy (38.7% vs. 12.7% and 6.9%) (Figure 1C). The mutations induced by Cas9 protein from TaKaRa included insertions and deletions of sizes ranging from 1 to 38 bp (Figure 1D). Interestingly, we also detected a substantial decrease in the sgRNA level in this experiment (TaKaRa Cas9 was used) at the two-cell stage (Figure 1B). Differently from the injection of *Cas9* mRNA, no further degradation of sgRNA was observed in injection of Cas9 protein after the two-cell stage (Figure 1B). We chose the Cas9 protein from TaKaRa for the following experiments.

Next, we examined the effect of the ratio of Cas9 to sgRNA on the efficiency of genome editing. We found that with the *mCherry*-sgRNA amount constant (200 ng/μL), a twofold increase of Cas9 protein from 500 ng/μL (Cas9:*mCherry*-sgNRA = 2.5:1) to 1000 ng/μL (Cas9:*mCherry*-sgNRA = 5:1) could induce significantly higher mutation efficacy (26.5% vs. 48.5%) (Figure 1E). However, increasing the Cas9 amount further (7.5:1 and 10:1) failed to induce more mutations (Figure 1E). Compared to the uninjected control, injections of the first three Cas9/*mCherry*-sgNRA combinations (2.5:1, 5:1 and 7.5:1) had little effect on embryonic development, while injection of the last combination (10:1) led to significantly more embryo deformation (Figure S2). A similar mutation efficacy change happened when we kept the Cas9 protein amount constant (500 ng/μL) and the sgRNA amount variable. A fourfold increase of sgRNA from 25 ng/μL (Cas9:*mCherry*-sgNRA = 1:0.05) to 100 ng/μL (Cas9:*mCherry*-sgNRA = 1:0.2) could induce a nearly four-fold increase in mutation efficacy (5.8% vs. 22.3%), and increasing the sgRNA amount further (1:0.5 and 1:1) was unable to cause more mutations (Figure 1F). These results indicated that the best weight ratio of Cas9 to sgRNA in amphioxus genome editing was 5:1. Interestingly, this weight ratio corresponded to a 1:1 molar ratio, consistent with the finding that Cas9 protein and sgRNA form a RNP complex in a 1:1 molar ratio manner. Moreover, the embryos injected with 5:1 of Cas9 (1000 ng/μL) to mCherry-sgRNA (200 ng/μL) failed to emit red fluorescence at the three-gill slit stage, while in uninjected larvae, around 50% of them showed red fluorescence in their somites and notochords (Figure 1G). This result demonstrated a high mutation efficacy for this combination of the Cas9 and *mCherry*-sgRNA.

We then determined the dynamics of mutation efficiency along the development of embryos injected with the Cas9 and *mCherry*-sgRNA RNP complexes. We found no detectable mutation in injected unfertilized eggs, and less than 10% mutation efficacy at the two-cell stage (around 1 h post- fertilization, 1 hpf) (Figure 1I). By the 32-cell stage (around 2 hpf), around 43.9% of the target site had been mutagenized; at the late blastula stage, the mutation efficacy peaked (around 50%) (Figure 1H,I). Therefore, compared to TALENs [5], CRISPR/Cas9 could induce mutations in amphioxus much faster.

### 3.3. The Broad Feasibility of the CRISPR/Cas9 System in Amphioxus Genome Editing

**Figure 2:**
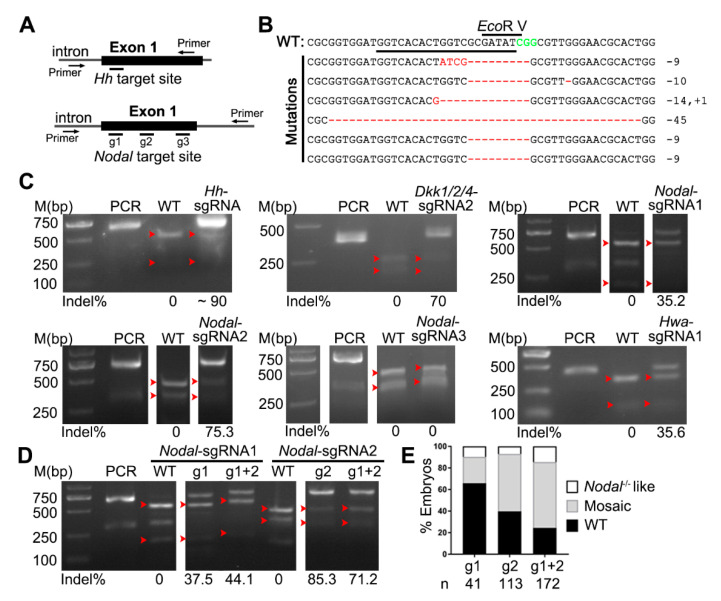
The broad feasibility of the CRISPR/Cas9 system in genome editing of amphioxus. (**A**) Schematic showing the position of the target site and the primers used for PCR amplicons in the *Hh* and *Nodal* loci in amphioxus. (**B**) Sanger sequencing results showing mutations (red) mediated by Cas9/sgRNA in an *Hh* allele. The wild-type (WT) reference sequence is shown at the top. The underlined sequence is the target site and CGG (green) is the PAM sequence. (**C**) Mutations were detected using a restriction enzyme digestion assay in the *Hh*, *Dkk1/2/4*, *Nodal* and *Hwa* loci. (**D**) Comparison of mutation efficacies between single-sgRNA and dual-sgRNAs injections in the *Nodal* locus. (**E**) Phenotypic evaluation of embryos injected with single-sgRNA or dual-sgRNAs targeting the *Nodal* gene. Two kinds of phenotypes were counted: embryos of the ‘*Nodal*^−/−^-like’ phenotype exhibited left isomerism morphology resembling embryos lacking Nodal signaling, and embryos of the ‘mosaic’ phenotype showed left-right defects in positioning of some organs. The number (n) of embryos evaluated is shown for each condition. In panels C and D, PCR denotes the PCR products not treated with the restriction enzyme, WT denotes digestion of PCR products from control (uninjected) embryos with the restriction enzyme, red arrowheads indicate bands released by restriction enzyme digestion and the induced mutation ratios (estimated as percentages of uncut PCR products) are labeled under each gel image.

To test whether the Cas9/sgRNA system was of broad spectrum in genome editing in amphioxus, we synthesized eight other sgRNAs targeting coding sequences of *Hedgehog* (*Hh*) (one), *Dkk1/2/4* (two), *Hwa* (two) and *Nodal* (three) genes (Figure 2A, Figure S3A) and injected each of them with the TaKaRa Cas9 protein into amphioxus embryos. Among these genes, function of the *Hh* gene has been clarified using the TALEN method [18], the role of *Nodal* has been addressed by a chemical inhibitor [19] and those of *Dkk1/2/4* and *Hwa* genes have never been studied. We detected mutations in embryos injected with *Hh*-sgRNA, *Dkk1/2/4*-sgRNA1, *Dkk1/2/4*-sgRNA2, *Hwa*-sgRNA1, *Nodal*-sgRNA1 and *Nodal*-sgRNA2 at efficacies ranging from 18.4% to 90% (Figure 2B,C, Figure S3C). By contrast, we detected no mutations for *Hwa*-sgRNA2 and *Nodal*-sgRNA3 using the restriction enzyme digestion assay (Figure 2C, Figure S3C) and DNA sequencing (Figure S3B). Moreover, we observed specific phenotypes in embryos injected with *Hh*-sgRNA, *Nodal*-sgRNA1 and *Nodal*-sgRNA2 (Figure S3D,E). These phenotypes (left isomerism and curled tail for *Hh*-sgRNA and right isomerism for *Nodal*-sgRNAs) were similar to those reported in embryos deficient of the *Hh* gene [18] or Nodal signaling activity [19]. We also injected *Nodal*-sgRNA1 and *Nodal*-sgRNA2 simultaneously and found that it created more embryos of the Nodal-deficient phenotype than single-sgRNA injections (Figure 2E), although mutation efficacies at each site were similar (Figure 2D).

### 3.4. Improvement of CRISPR/Cas9-Mediated Mutation Efficiency in Amphioxus

**Figure 3:**
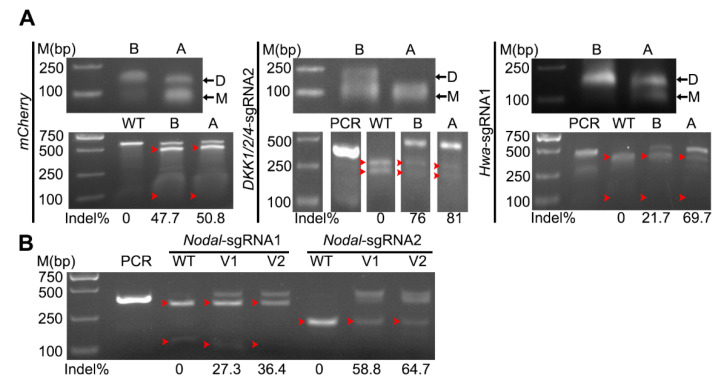
Improvement of mutation efficiency of Cas9/sgRNA in amphioxus. The induced mutation ratios shown under each gel image were estimated by either a T7 endonuclease assay (for *mCherry*-sgRNA) or a restriction endonuclease assay (for sgRNAs targeting the *Dkk1/2/4*, *Nodal* and *Hwa* genes). Red arrowhead indicates bands released by endonuclease digestions. PCR, PCR products not treated with the restriction enzyme; WT, digestion of PCR products from control (uninjected) embryos with the restriction enzyme. (**A**) Monomerization of sgRNAs (top) and its effect on mutation efficiency (bottom)**.** sgRNA was monomerized using a denaturing step. B, before denaturation; A, after denaturation; D, dimer, M, monomer. (**B**) Comparison of mutation efficacies between version 1 (V1) and version 2 (V2) sgRNAs targeting the *Nodal* gene.

The in vitro-synthesized sgRNAs existed in two bands in agarose gel (Figure 3A), which probably resulted from dimerization or secondary structure. We referred to the short one (~100bp) as the monomer and the long one (~200bp) as the dimer for simplicity, according to a previous report [12]. Denaturation of sgRNA by heating and quick cooling could change the dimer to the monomer and increase mutation efficacy [12]. To test this in amphioxus, we denatured the synthesized sgRNAs before injection and compared their efficacies with the undenatured ones. The treatment could efficiently reduce the proportion of dimers in all three tested sgRNAs (*mCherry*-sgRNA, *Dkk1/2/4*-sgRNA2 and *Hwa-*sgRNA1) (Figure 3A), and induce more mutations at their target sites than the undenatured ones (Figure 3A). Especially at the site targeted by *Hwa*-sgRNA1, mutation efficacy was increased dramatically from 21.7% to 69.7%. Interestingly, this high level of efficacy increment correlated well with highly efficient turnover of *Hwa*-sgRNA1 from dimer to monomer by the treatment (from less than 5% to around 40%) (Figure 3A). This result demonstrated that predenaturation of sgRNAs before mixing them with Cas9 protein could increase mutation efficacies in amphioxus, which was especially true for sgRNAs, which tend to form dimers.

Although the sgRNA backbone used in the experiments above is already optimized [11] and has been widely used in many previous studies, a recent study showed that inclusion of a 5 bp duplex extension and a T-to-C mutation at position 4 of the continuous sequence of thymines in the above-mentioned backbone could further improve genome editing efficiency in cells [12]. To test whether this was a similar case in amphioxus, we synthesized two *Nodal*-sgRNAs of the newly updated backbone (named as version 2) and compared their efficacies to those of the original ones (named as version 1). The result showed that the two version 2 sgRNAs could both induce slightly more mutations than the original ones (36.4% vs. 27.3% for *Nodal*-sgRNA1, 64.7% vs. 58.8% for *Nodal*-sgRNA2) (Figure 3B).

## 4. Conclusions

In summary, we have successfully introduced the CRISPR/Cas9 system into amphioxus and demonstrated that it was able to induce targeted mutations highly efficiently in this emerging model organism. Considering the simplicity of the CRISPR/Cas9 system, our work will certainly motivate more researchers to use the genome editing method to study gene function in amphioxus. In addition, we found that nearly half of the tested sgRNAs (4/9) gave specific phenotypes among the injected embryos. This indicates a great potential for using it to knockdown gene function in amphioxus embryos. Moreover, our work also opens possibility for exploring other CRISPR/Cas9-based technologies such as epigenetic modulation, inducible regulations and base editing [20] in amphioxus.

## Supplementary Materials

The following are available online at https://www.mdpi.com/2073-4425/11/11/1311/s1, Figure S1: Cas9/sgRNA ribonucleoprotein complex induces site-specific mutations at *mCherry* locus in *mylz2*-mCherry transgenetic amphioxus, Figure S2: Impact of different concentration combinations of Cas9/sgRNA on amphioxus embryonic development, Figure S3: The broad feasibility of CRISPR/Cas9 system in genome editing of amphioxus, Table S1: Primers for construction of sgRNA vectors (V1 or V2), Table S2: The sequences of different sgRNA structure used in this study, Table S3: Primers used for PCR-based genotyping.
