# Supplementary material for: Application of CRISPR/Cas9 Nuclease in Amphioxus Genome Editing
Source: Genes (Basel). 2020 Nov 5;11(11):1311. doi: 10.3390/genes11111311 (PMC7694359; doi:10.3390/genes11111311)
Supplement: Supplementary file 1 [file genes-11-01311-s001.pdf]

Supplementary Figures and Tables

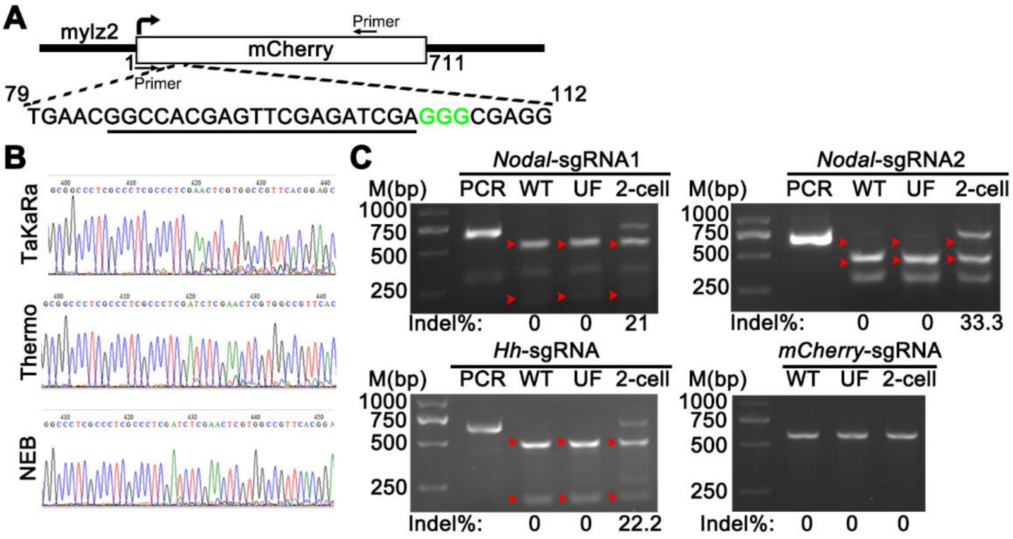

Supplementary Figure S1. Cas9/sgRNA ribonucleoprotein complex induces site-specific mutations at *mCherry* locus in *mylz2-mCherry* transgenetic amphioxus. (A) Schematic showing the position of the target site and the primers used for PCR amplication in the *mCherry* gene. Sequence underlined is the target site and CGG (green) is the PAM sequence. (B) Sanger sequencing of *mCherry* locus from embryos injected with *mCherry*-sgRNA and Cas9 nuclease from three companies. (C) Enzyme digestion showing mutation induced by injecting Cas9 mRNA and corresponding sgRNAs at 2-cell stage.

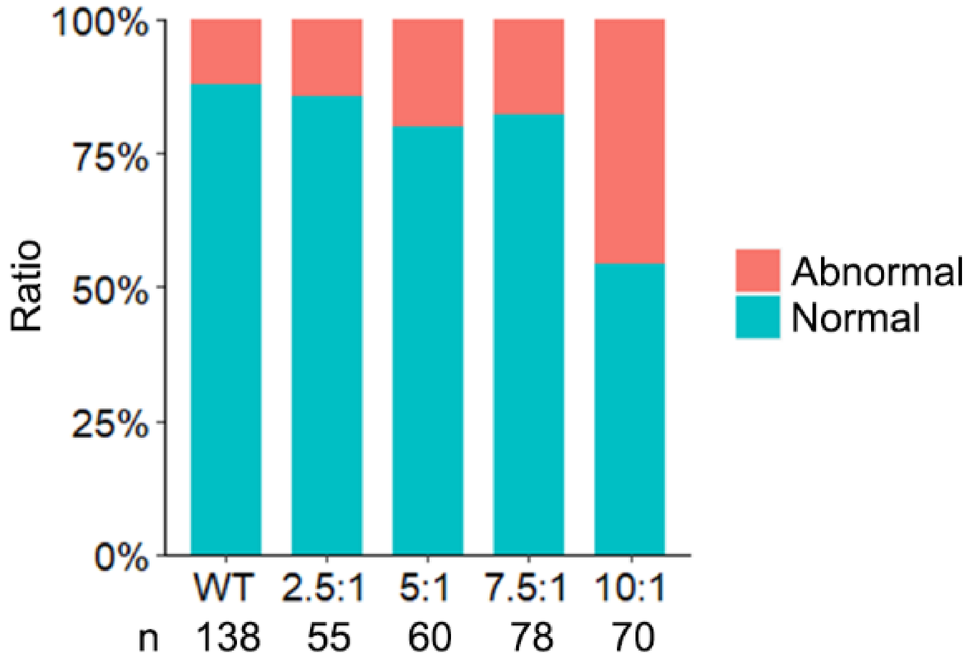

Supplementary Figure S2. Impact of different concentration combinations of Cas9/sgRNA on amphioxus embryonic development. Embryos were examined 3 days post injection (at 3-gill slit stage). n represents numbers of larvae examined.

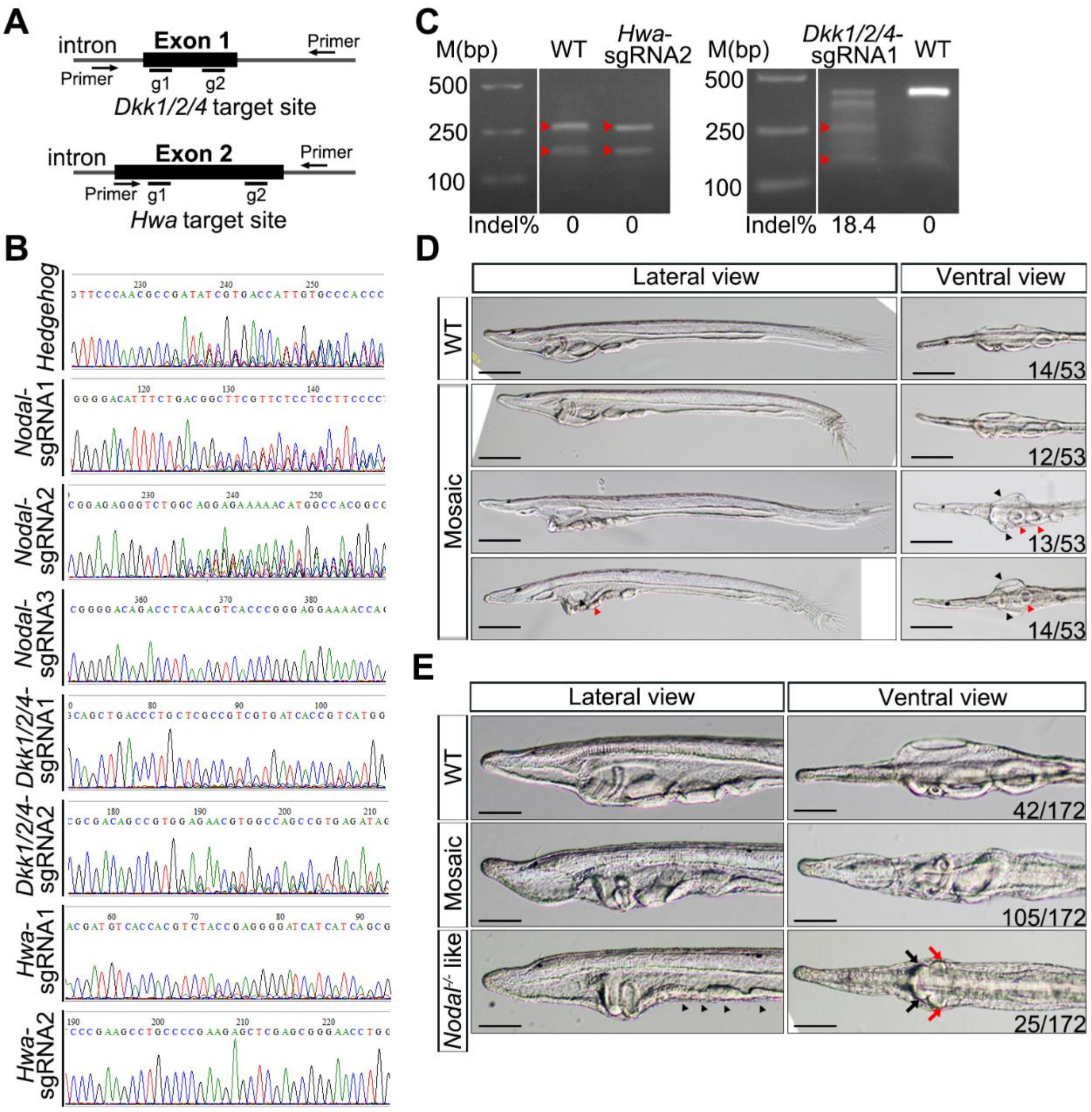

Supplementary Figure S3. The broad feasibility of CRISPR/Cas9 system in genome editing of amphioxus.

(A) Schematic showing the target sites and the primers used for PCR amplicons in the *Dkk1/2/4* and *Hwa* loci in amphioxus. (B) Sanger sequencing of amplicons of embryos injected with Cas9 nuclease and corresponding sgRNAs. (C) Mutation detection in embryos injected with Cas9 nuclease and *Hwa*-sgRNA2 (by restriction enzyme digestion assay) or *Dkk1/2/4*-sgRNA1 (by T7EI cleavage assay). Red arrowheads indicate bands released by endonuclease digestion. The induced mutation ratios are labeled under each gel image. (D) Representative phenotypes observed in 2-day larvae injected with Cas9/*Hh*-sgRNA RNP complexes. Among the 53 larvae examined, 14 were normal (WT) and 39 showed different level of similarities to $Hh^{-/-}$ mutants (mosaic); out of the 39 'mosaic' larvae, 12 developed a twisted tail (top), 13 left isomerism morphology (middle), and 14 showed both of the former phenotypes (bottom). It should be noted that none of these larvae lacked mouth like $Hh^{-/-}$ mutants.

Black arrowheads indicate the two symmetric mouths and red arrowheads indicate the ventralized gill slits that normally appear in the right side. (E) Representative phenotypes observed in 2-day larvae injected with Cas9 and mixture of *Nodal*-sgRNA1 and *Nodal*-sgRNA2 (version 1). Only the anterior portion of the larvae is shown, because no defects was observed in their posterior ends. Black arrowheads demarcate the ventralized and improperly developed gill slits, black arrows indicate the symmetric endostyles, and red arrows indicates the symmetric club-shaped gland. Scale bars in D and E are respectively 50 μm and 25 μm.

Table S1 Primers for construction of sgRNA vectors (V1 or V2)

Targeting sites	Oligos (5’ to 3’)
mCherry	Forward: taggCCACGAGTTCGAGATCGA<br>Reverse: aaacTCGATCTCGAACTCGTGG
Hedgehog	Forward: taggTCACACTGGTCGCGATAT<br>Reverse: aaacATATCGCGACCAGTGTGA
Dkk1/2/4-sgRNA1	Forward: taggTGATCACGACGGCGAGCA<br>Reverse: aaacTGCTCGCCGTCGTGATCA
Dkk1/2/4-sgRNA2	Forward: taggCGACAGCCGTGGAGAACG<br>Reverse: aaacCGTTCTCCACGGCTGTCG
Nodal-sgRNA1	Forward: taggGGACATTTCTGACGGCTT<br>Reverse: aaacAAGCCGTCAGAAATGTCC
Nodal-sgRNA2	Forward: taggCGGAGAGGGTCTGACGCT<br>Reverse: aaacAGCGTCAGACCCTCTCCG
Nodal-sgRNA3	Forward: taggACAGACCTCAACGTCACC<br>Reverse: aaacGGTGACGTTGAGGTCTGT
Hwa-sgRNA1	Forward: taggTGTCACCACGTCTACCGA<br>Reverse: aaacTCGGTAGACGTGGTGACA
Hwa-sgRNA2	Forward: taggTTCCCGCTCGAGCTCTTC<br>Reverse: aaacGAAGAGCTCGAGCGGGAA
pT7-gRNA-F1	GGAATTAATACGACTCACTA
Dr-gRNA-R1	AAAGCACCGACTCGGTGCCAC
universal primer	AAAGCACCGACTCGGTGCCACTTTTTCAAGTTGATAACGGACTAGCCTTATTTAAACTTGC<br>TATGCTGTTTCCAGCATAGCTCTTAAAC
BfNodal-gRNA1	GGAATTAATACGACTCACTATAGGGGACATTTCTGACGGCTTGTTTAAGAGCTATGCTGG

BfNodal-gRNA2	GGAATTAATACGACTCACTATAGGCGGAGAGGGTCTGACGCTGTTTAAGAGCTATGCTGG
mCherry-gRNA1	GGAATTAATACGACTCACTATAGGCCACGAGTTCGAGATCGAGTTTAAGAGCTATGCTGG

Note: red letters denote the target sequences

Table S2 The sequences of different sgRNA structure used in this study

V1	$(N)_{20}$GTTTAGAGCTAGAAATAGCAAGTTAAAATAAGGCTAGTCCGTTATCAACTTGAAAAAGTGGCACCGAGTCGGTGCGGATC
V2	$(N)_{20}$GTTTAAGAGCTATGCTGGAAACAGCATAGCAAGTTTAAATAAGGCTAGTCCGTTATCAACTTGAAAAAGTGGCACCGAGTCGGTGCTTT

Table S3 Primers used for PCR-based genotyping

Primers	Forward primer (5' to 3')	Reverse primer (5' to 3')
mCherry	cggccgTGGTGAGCAAGGGCGAGGAG	TCTTGACCTCAGCGTCGTAGTG
Hedgehog	GCCGCTTTCTGCCTTGCTTCA	GCGAGTAATCCGTCCGTTGA
Dkk1/2/4	AGGATCTTCCCGAAACTCGC	AAGACGTACACCGGAAAGCC
Nodal-1	CCGCCTTGCCTTTGTCTTTC	CACTGGTAGAAACGTCGTCCA
Nodal-2	GGCAGGCCGAGACCAACACC	AGTGCGAAACTCCTGACGGTGTCG
Hwa	GAACAGGTGACTCAAACCCAAAG	TCTGGTCTCTGTGAAATGGCTTA

Note: The lowercase in *mCherry* forward primer means the *Eag*I recognition sequence. The *Nodal*-2 Primers were only used in Fig. S4B for the reason that the *Nodal*-1 Primers were nonspecific at that experiment.

Table S4 Primers used for qPCR

qPCR Primers	Forward primer (5' to 3')	Reverse primer (5' to 3')
mCherry-sgRNA-RT	CACGAGTTCGAGATCGAGTTTT	CCGACTCGGTGCCACTTTTT
(WH)pXT7-hCas9-RT	AGGAGGACATCCAGAAAGCAC	TTCTCGGGCTTATGCCTTCC
Gapdh	GGTGGAAAGGTCCTGCTCTC	CTGGATGAAAGGGTCGTTAATGG
